# Identification of new genes associated with breast and ovarian cancer risk. Advances of BCAC, CIMBA and OCAC

**DOI:** 10.1186/1897-4287-9-S2-A1

**Published:** 2011-06-01

**Authors:** A Jakubowska, J Gronwald, K Jaworska, K Durda

**Affiliations:** 1 International Hereditary Cancer Center, Pomeranian Medical University, Szczecin, Poland

## 

Breast and ovarian cancers belong to the most common malignancies diagnosed in women. The major inherited susceptibilities to breast and ovarian cancers are germline mutations in either *BRCA1* or *BRCA2* which however, explain only small number of breast and ovarian cancer cases. Data suggest that majority breast and ovarian cancers are caused by low or moderate penetrance gene mutations. Identification of such mutations provides cancer risk assessment and will help in prophylactic, early diagnosis and treatment.

In 2005 three international multidisciplinary consortia have been initiated: **Breast Cancer Association Consortium** (BCAC), **Consortium of Investigators of Modifiers of BRCA1/2** (CIMBA) and **Ovarian Cancer Association Consortium** (OCAC) which are forums of investigators from centers over the world, including International Hereditary Cancer Center in Szczecin. The aim of these consortia’s is to combine data from many studies, to provide a reliable assessment of the breast and ovarian cancer risks associated with different genetic and environmental factors, and to identify potential modifiers of cancer risk in carriers of BRCA1 and BRCA2 mutation.

**BCAC** includes 54 centers (table 1) over the world and focuses on identification of genes associated with breast cancer risk.

**Table 1:** Centers collaborating in BCAC

Name	Acronym	Country
Australian Breast Cancer Family Study	ABCFS	Australia
Amsterdam Breast Cancer Study	ABCS	Netherlands
Australian Breast Cancer Tissue Bank	ABCTB	Australia
Asia Cancer Program	ACP	Thailand
Bavarian Breast Cancer Cases and Controls	BBCC	Germany
British Breast Cancer Study	BBCS	UK
Breast Cancer In Galway Genetic Study	BIGGS	Ireland
Breast Cancer Study of the University Clinic Heidelberg	BSUCH	Germany
CECILE Breast Cancer Study	CECILE	France
Copenhagen General Population Study	CGPS	Denmark
Spanish National Cancer Centre Breast Cancer Study	CNIO-BCS	Spain
California Teachers Study	CTS	USA
ESTHER Breast Cancer Study	ESTHER	Germany
ICR Familial Breast Cancer Study	FBCS	UK
German Consortium for Hereditary Breast & Ovarian Cancer	GC-HBOC (GFBCS)	Germany
Gene Environment Interaction and Breast Cancer in Germany	GENICA	Germany
Genetic Epidemiology Study of Breast Cancer by Age 50	GESBC	Germany
Hannover Breast Cancer Study	HABCS (HBCS)	Germany
Helsinki Breast Cancer Study	HEBCS	Finland
Hannover-Minsk Breast Cancer Study	HMBCS	Belarus
Hannover-Ufa Breast Cancer Study	HUBCS	Russia
Karolinska Breast Cancer Study	KARBAC	Sweden
Kuopio Breast Cancer Project	KBCP	Finland
kConFab/AOCS	kConFab/AOCS	Australia
Leuven Multidisciplinary Breast Centre	LMBC	Belgium
Mammary Carcinoma Risk Factor Investigation	MARIE	Germany
Milan Breast Cancer Study Group	MBCSG	Italy
Mayo Clinic Breast Cancer Study	MCBCS	USA
Melbourne Collaborative Cohort Study	MCCS	Australia
Multi-ethnic Cohort	MEC	USA
Memorial Sloan-Kettering Cancer Center	MSKCC	USA
Mexican Case Control Study of Breast Cancer	MXCCS	Mexico
Malaysian Breast Cancer Genetic Study	MYBRCA	Malaysia
Norwegian Breast Cancer Study	NBCS	Norway
Northern California Breast Cancer Family Registry	NC-BCFR	USA
Nurses Health Study	NHS	USA
Nigerian Breast Cancer Study	NGBCS	Nigeria
Oulu Breast Cancer Study	OBCS	Finland
Ontario Familial Breast Cancer Registry	OFBCR	Canada
Leiden University Medical Centre Breast Cancer Study	ORIGO (LUMCBCS)	Netherlands
NCI Polish Breast Cancer Study	PBCS	Poland
Prospective Study of Outcomes in Sporadic Versus Hereditary Breast Cancer	POSH	UK
Rotterdam Breast Cancer Study	RBCS	Netherlands
Singapore and Sweden Breast Cancer Study	SASBAC	Sweden
Sheffield Breast Cancer Study	SBCS	UK
Study of Epidemiology and Risk factors in Cancer Heredity	SEARCH	UK
Seoul Breast Cancer Study	SEBCS (SBCP)	Korea
**IHCC-Szczecin Breast Cancer Study**	**SZBCS**	**Poland**
IARC-Thai Breast Cancer Study	TBCS	Thailand
Taiwanese Breast Cancer Study	TWBCS	Taiwan
UCI Breast Cancer Study	UCIBCS	USA
UK Breakthrough Generations Study	UKBGS	UK
US Three State Study	US3SS	USA
US Radiologic Technologists Study	USRTS	USA

Currently, BCAC database includs demographic, clinical and epidemiological data from 73,000 breast cancer patients and 80,000 unaffected women. Up to now, 125 genetic alterations (SNPs) localized in different genes have been examined. These SNPs have been selected based on its positive association with breast cancer risk detected in preliminary analyses or Genome-Wide Association Study (GWAS). Results have been presented in 12 manuscripts published in high impact journals, e.g. J Natl Cancer Inst, Nature and Nat Genet (http://www.srl.cam.ac.uk/consortia/bcac/pubs/pubs.html).

Ten SNPs have been found to be associated with breast cancer risk overall or by clinical and pathological characteristics (table 2).

**Table 2:** BCAC SNPs associated with breast cancer risk overall or by clinical and pathological characteristics

Gene / SNP	Breast cancer risk	BCAC manuscript
CASP8 D302H	CG v. GG: OR 0.89, 95% CI 0.85-0.94 CC v. GT: OR 0.74, 95% CI 0.62-0.87	*Cox et al.*, *Nat Genet 2007*

FGFR2, rs2981582 TNRC9, rs3803662 MAP3K1, rs889312 LSP, rs3817198 8q24, rs13281615	OR_hom_ 1.63, 95% CI 1.53-1.72, p=4.1 × 10^-76^ OR_hom_ 1.39, 95% CI 1.25-1.45, p=1.4 × 10^-36^ OR_hom_ 1.27, 95% CI 1.19-1.36, p=4.1 × 10^-20^ OR_hom_ 1.17, 95% CI 1.08-1.25, p=4.1 × 10^-9^ OR_hom_ 1.18, 95% CI 1.10-1.25, p=4.1 × 10^-12^	*Easton et al.*, *Nat Genet 2007*

FGFR2, 2981582 8q24, 13281615 TNRC9, rs3803662	ER positive:p=10^-13^ PGR positive: p=10^-5^ low grade: p=10^-8^ metastases: p=0.013 ER positive: p=0.001 PGR positive: p=0.011 low grade: p=10^-4^ ER negative: OR 1.14, 95% CI 1.09-1.21	*Garcia-Closas et al.*, *PLoS Genet 2008*

AKAP9 M463I	TT v. GG: OR 1.17, 95% CI 1.08-1.27, p=0.0003 familial cases: OR 1.27, 95% CI 1.12-1.45, p=0.0003 TT v. GT: OR 1.10, 95% CI 1.04-1.17, p=0.001 familial cases: OR 1.16, 95% CI 1.06-1.27, p=0.001	*Frank et al.*, *JNCI 2008*

3p24, rs4973768 17q23, rs6504950	OR_per-allele_ 1.11, 95% CI 1.08-1.13, p=4.1 × 10^-23^ OR_per-allele_ 0.95, 95% CI 0.92-0.97, p=1.4 × 10^-8^	*Ahmed et al.*, *Nat Genet 2009*

2q35, rs13387042	OR _per-allele_ 1.12, 95% CI 1.09 -1.15; p_trend_ 1.0 × 10^-19^ (European Caucasian) ER positive: OR 1.14, 95% CI 1.10-1.17; p=10^-15^ ER negative: OR 1.10, 95% CI 1.04-1.15; p=0.0003 PGR positive: OR 1.15, 95% CI 1.11-1.19; p=5 × 10^-14^ PGR negative: OR 1.10, 95% CI 1.06-1.15; p=0.00002	*Milane et al.*, *JNCI 2009*

CIMBA database contains demographic, clinical and epidemiological data from 15,700 BRCA1 and 8,600 BRCA2, mutation carriers including 12,700 breast cancer patients, 2,500 ovarian cancer patients and 9,100 unaffected patients from 42 centers over the world (table 3).

**Table 3:** Centers collaborating in CIMBA

Study	Acronym	Country
Breast Cancer Family Registry	BCFR	USA/Australia
Baltic Familial Breast Ovarian Cancer Consortium	BFBOCC	Latvia/Lithuania
BRCA-gene mutations and breast cancer in South African women	BMBSA	South Africa
Rigshospitalet	CBCS	Denmark
Spanish National Cancer Centre	CNIO	Spain
CONsorzio Studi ITaliani sui Tumori Ereditari Alla Mammella	CONSIT TEAM	Italy
German Cancer Research Center	DKFZ	Germany
Genen Omgeving studie van de werkgroep Hereditiair Borstkanker Onderzoek Nederland	DNA HEBON	Netherlands
Epidemiological Study of Familial Breast Cancer	EMBRACE	UK
Fox Chase Cancer Center	FCCC	USA
German Familial Breast Group	GC-HBOC	Germany
Genetic Modifiers of cancer risk in BRCA1/2 mutation carriers	GEMO	France/Greece/USA
Georgetown University	GEORGETOWN	USA
Gynecologic Oncology Group	GOG	USA
Hospital Clinico San Carlos	HCSC	Spain
Helsinki Breast Cancer Study	HEBCS	Finland
Study of Genetic Mutations in Breast and Ovarian Cancer patients in Hong Kong and Asia	HRBCP	Hong Kong
Molecular Genetic Studies of Breast- and Ovarian Cancer in Hungary	HUNBOCS	Hungary
Institut Català d'Oncologia	ICO	Spain
**International Hereditary Cancer Centre**	**IHCC**	**Poland**
Iceland Landspitali - University Hospital	ILUH	Iceland
INterdisciplinary HEalth Research Internal Team BReast CAncer susceptibility	INHERIT	Canada
Istituto Oncologico Veneto	IOVHBOCS	Italy
Kathleen Cuningham Consortium for Research into Familial Breast Cancer	KCONFAB	Australia/New Zealand
Korean Hereditary Breast Cancer Study	KOHBRA	Korea
Modifiers and Genetics in Cancer	MAGIC	USA
Mayo Clinic	MAYO	USA
Modifier Study of Quantitative Effects on Disease	MOD SQUAD	USA
Memorial Sloane Kettering Cancer Center	MSKCC	USA
General Hospital Vienna	MUV	Austria
National Cancer Institute	NCI	USA
National Israeli Cancer Control Center	NICCC	Israel
N.N. Petrov Institute of Oncology	NNPIO	Russia
Ontario Cancer Genetics Network	OCGN	Canada
The Ohio State University Comprehensive Cancer Center	OSU CCG	USA
Odense University Hospital	OUH	Denmark
Università di Pisa	PBCS	Italy
South East Asian Breast Cancer Association Study	SEABASS	Malaysia/Singapore
Sheba Medical Centre	SMC	Israel
Swedish Breast Cancer Study	SWE-BRCA	Sweden
University of California Irvine	UCI	USA
University of California San Francisco	UCSF	USA
UK and Gilda Radner Familial Ovarian Cancer Registries	UKGRFOCR	UK/USA
University of Pennsylvania	UPENN	USA
Cedars-Sinai Medical Center	WCRI	USA

Results have been presented in 9 manuscripts published in high impact journals, e.g. Am. J Hum Genet, Hum Mol Genet (http://www.srl.cam.ac.uk/consortia/cimba/pubs/pubs.html).

Six SNPs have been found to be associated with breast cancer risk (table 4).

**Table 4:** SNPs associated with breast cancer risk in BRCA1/2 carriers

Gene / SNP	Breast cancer risk	CIMBA manuscript
RAD51, rs11683487	BRCA2: HR_hom_ 3.18, 95% CI 1.39-7.27, p=0.0007	*Antoniou et al. AJHG 2007*

TNRC9, rs3803662 FGFR2, rs2981582 MAP3K1, rs889312	BRCA1/2: HR 1.13, 95% CI: 1.06-1.20, p_trend_= 5 × 10^-5^ BRCA2: HR 1.32, 95% CI: 1.20-1.45, p_trend_=1.7 × 10^-8^ BRCA2: HR 1.12, 95% CI: 1.02-1.24, p_trend_=0.02	*Antoniou et al. AJHG 2008*

2q35, rs13387042 LSP1, rs3817198 2q35, rs13387042	BRCA1: HR 1.14, 95% CI: 1.04-1.25, p=0.0047 BRCA2: HR 1.16, 95% CI: 1.07-1.25, p_trend_=2.8 × 10^-4^ HR 1.18 95% CI: 1.04-1.33, p=0.0079	*Antoniou et al. Hum Mol Genet 2008*

**OCAC** includes 24 centers over the world and focuses on identification of genes that may be related to the risk of ovarian cancer (table 5).

**Table 5:** Centers collaborating in OCAC

Name	Acronym	OCAC Acronym	Country
Australian Ovarian Cancer Study and Australian Cancer Study (Ovarian Cancer)	AOCS/ACS	AUS	Australia
Bavarian Ovarian Cancer Cases and Controls	BOCC	BAV	Germany
Belgian Ovarian Cancer Study	BELOCS	BEL	Belgium
Connecticut Ovary Study	(none)	CON	USA
Diseases of the Ovary and their Evaluation Study	DOVE	DOV	USA
German Ovarian Cancer Study	GOCS	GER	Germany
Hawaii Ovarian Cancer Study	(none)	HAW	USA
Hannover-Jena Ovarian Cancer Study	HJOCS	HJO	Germany
Hannover-Minsk Ovarian Cancer Study	HMOCS	HMO	Germany
Helsinki Ovarian Cancer Study	HOCS	HOC	Finland
Hormones and Ovarian Cancer Prediction	HOPE	HOP	USA
**Polish Ovarian Cancer Study**	**POCS**	**JAC**	**Poland**
Women's Cancer Research Institute (Cedars-Sinai Medical Center)	WCRI	LAX	USA
The Danish Malignant Ovarian Tumour Study	MALOVA	MAL	Denmark
Mayo Clinic Ovarian Cancer Case Control Study	(none)	MAY	USA
Melbourne Collaborative Cohort Study	MCCS	MCC	Australia
Memorial Sloan Kettering Cancer Center Gynecology Tissue Bank	MSKGTB	MSK	USA
North Carolina Ovarian Cancer Study	NCOCS	NCO	USA
New England-based Case-Control Study of Ovarian Cancer	NECC	NEC	USA
Nurses Health Study	NHS	NHS	USA
New Jersey Ovarian Cancer Study	NJOCS	NJO	USA
Nijmegen Polygene Study & Nijmegen Biomedical Study	POLYGENE	NTH	Netherlands
Prognostic Factors in Epithelial Ovarian Cancer	EOC	NTX	Netherlands
Ovarian Cancer in Alberta and British Columbia Study	OVAL-BC	OVA	Canada
NCI Ovarian Case-Control Study in Poland	NCI-OCS	POL	Poland
Roswell Park Cancer Institute Cases	RPCI	RPX	USA
UK SEARCH Ovarian Cancer Study	SEARCH	SEA	UK
Southampton Ovarian Cancer Study	(none)	SOC	UK
Genetic Epidemiology of Ovarian Cancer	GEOCS	STA	USA
Tampa Bay Ovarian Cancer Study	TBOCS	TBO	USA
Familial Ovarian Tumour Study	FOTS	TOR	Canada
UC Irvine Ovarian Cancer Study	(none)	UCI	USA
UK Ovarian Cancer Population Study	UKOPS	UKO	UK
Los Angeles County Case-Control Studies of Ovarian Cancer	LAC-CCOC	USC	USA

Currently, OCAC database includs demographic, clinical and epidemiological data from 22,000 ovarian cancer patients and 18,000 unaffected women. Several genetic alterations (SNPs) localized in different genes have been examined. Results have been presented in 13 manuscripts published in high impact journals, e.g. Nat Genet, Cancer Res, In J Cancer (http://www.srl.cam.ac.uk/consortia/ocac/pubs/pubs.html).

Seven SNPs have been found to be associated with ovarian cancer risk overall or by clinical and pathological characteristics (table 6).

**Table 6:** SNPs associated with ovarian cancer risk

Gene / SNP	Ovarian cancer risk	OCAC manuscript
CDKN2A, rs3731257 CDKN1B, rs2066827	OR 0.91, 95% CI 0.85-0.98, p=0.008 OR 0.93, 95% CI 0.87-0.995, p=0.036	*Gayther et al.*, *Cancer Res 2007*

TP53, 23 SNPs: rs2287498 rs12951053	OR_per-allele_ 1.30, 95% CI 1.07-1.57 OR_per-allele_ 1.19, 95% CI 1.01-1.38	*Schildkraut et al.*, *Cancer Res 2009*

NMI, rs11683487	OR 0.89, 95% CI 0.80-0.99, p=0.032	*Quaye et al.*, *BJC 2009*

CYP3A4, rs2740574	OR_hom_ 2.81, 95% CI 1.20-6.56, p=0.017	*Pearce et al.*, *BJC 2009*

9p22.2, rs3814113	OR 0.82, 95% CI 0.79-0.86, p=5.1×10^-19^ serous tumors: OR 0.77, 95% CI 0.73-0.81, p=4.1×10^-21^	*Song et al.*, *Nat Genet 2009*

